# Bacterial Toxins

**Published:** 1903-03-21

**Authors:** 


### The Hospital.

A Journal of

The Medical Sciences and Hospital Administration.

Vol. XXXIII.—No. 860. Edited by Sir Henry Burdett, K.C.B., and Solomon C. Smith. M.D., M.R.C.P. [March 21, 1903.

### Bacterial Toxins.

The highly important paper by Dr. Macfadyen, of the Jenner Institute of Preventive Medicine, which was communicated to the Royal Society by Lord Lister, and read at the meeting on the 12th instant, seems to justify a hope that we are on the threshold of practical applications of bacterial toxins for the prevention of many varieties of disease. The paper was founded upon researches carried on at the Institute, and was limited to an account of the results actually obtained with the bacillus of typhoid fever; but these results appear, as far as can at present be judged, to rest upon principles likely to be of widely extended application. It is well known that injections of pathogenetic bacteria into living animals are productive of very uncertain consequences; the bacteria may be more or less active as regards their power of multiplication, and more or less virulent in their effects; while the condition of the animal may be such as either to favour or to hinder their reproduction, or such as either to resist or to succumb to their influence. As the result of experimental exposure of typhoid bacilli to the temperature of liquid air, Dr. Macfadyen found that their vitality remained unimpaired when they were restored to a normal temperature, but that while frozen they might be crushed in such a way as to destroy their life and power of multiplication, leaving their toxic properties unimpaired. In such crushed bacilli, therefore, he obtained the typhoid poison in a state of high concentration, but deprived of the power of indefinite increase by multiplication in the body of the recipient, and therefore capable of administration in an exact and regulated dosage. Experiments with the material thus obtained showed that the blood of a living animal, to which small and repeated doses had been administered, became powerfully antitoxic and bactericidal, and furnished an effective antidote to injections which otherwise would have been fatal. In subsequent experiments it was demonstrated that the antidote would neutralise, and, so to speak, overtake, the typhoid poison even if not administered until after the latter had begun to do its work; thus justifying the belief that it will furnish not only an effective prophylactic against the disease, but also an effective remedy against it even after the symptoms have become declared. It is reasonable to expect that the action of other pathogenetic bacteria will be identical with, or at least closely analagous to, that of the bacillus of typhoid, and that a similar method of preparation and of administration will before long be rendered available in the case of many other diseases and forms of infection. When experimentation on the lower animals is sufficiently advanced, it will become justifiable to apply approved methods of administration to mankind; and we may be sure that the spirit which produced volunteers for researches into the production of malaria and of yellow fever will not be wanting when the time comes for its display. We would even venture to suggest to the officials and committees of the various anti-vivisection societies that they might for once take part in the advancement of knowledge; and that, if they would offer themselves as subjects for experiment, they might not only be the indirect means of saving the lives of myriads of their fellow-creatures, but they would certainly effect the (in their sight) more important object, of preserving a score or two of rats, rabbits, or guinea-pigs from the puncture of the injection syringe. Surely they will find the temptation irresistible, and will flock to the Jenner Institute in order to place their bodies at the disposal of Dr. Macfadyen and his colleagues.

Contemporaneously with the publication of Dr. Macfadyen's results, we hear through Reuter of Professor Behring's account of his experiments with a tuberculosis serum of his manufacture, obtained by cultivating the bacillus and drying it *in vacuo*. The Professor is said to have lectured on the subject before the Medical Society of Vienna, and to have expressed himself hopefully, but with the reservation that the time had not yet come for the extension of his experiments from animals to the human subject. The memory of the disappointment caused by the premature publication of Professor Koch's sanguine expectations from tuberculin has not yet sufficiently died away to prepare the public mind for any enthusiastic reception of a treatment which seems to rest upon a very similar foundation; but it is practically impossible to doubt that victory over pathogenetic bacteria is in the air, and that its attainment is to be looked for upon the lines of an immunity, resting upon the same grounds as the natural immunity which is conferred, in so many cases, by an ordinary attack of the disease concerned. Neither at home nor abroad, however, should an increased prospect of success in encountering bacterial or parasitic diseases be accepted as an excuse, still less as a reason, for any neglect of the precautions on which immunity from disease in general must be founded.

